# A new animal model containing human SCARB2 and lacking *stat-1* is highly susceptible to EV71

**DOI:** 10.1038/srep31151

**Published:** 2016-08-08

**Authors:** An-Ting Liou, Szu-Yao Wu, Chun-Che Liao, Ya-Shu Chang, Chih-Shin Chang, Chiaho Shih

**Affiliations:** 1 Taiwan International Graduate Program (TIGP) in Molecular Medicine, National Yang-Ming University and Academia Sinica, Taipei, Taiwan; 2 Institute of Biomedical Sciences, Academia Sinica, Taipei, Taiwan; 3 Institute of Biochemistry and Molecular Biology, National Yang-Ming University, Taipei, Taiwan; 4 Institute of Microbiology and Immunology, National Yang-Ming University, Taipei, Taiwan

## Abstract

Enterovirus 71 (EV71) is a major threat to children worldwide. Children infected with EV71 could develop subclinical infection and hand-foot-and -mouth disease (HFMD). In severe cases, patients could develop encephalitis, paralysis, pulmonary edema, and death. A more user-friendly and robust animal model is essential to investigating EV71 pathogenesis. Here, we established a hybrid (hSCARB2^+/+^/*stat-1*^−/−^) mouse strain from crossbreeding SCARB2 transgenic and *stat-1* KO mice, and compared the susceptibilities to EV71 infection and pathogenesis between parental and hybrid mice. Virus-encoded VP1 protein can be detected in the streaking nerve fibers in brain and spinal cord. This hybrid mouse strain at 2-week-old age can still be infected with different genotypes of EV71 at 1000-fold lower titer via an ip route. Infected hybrid mice developed earlier onset of CNS disease, paralysis, and death at a higher incidence. These advantages of this novel model meet the urgent need from the scientific community in basic and preclinical research in therapeutics and pathogenesis.

Enterovirus 71 (EV71) was first identified in California in 1969^1^. It is a member of the *Picornaviridae* family, and is related to poliovirus^2^, hepatitis A virus (EV72)^3^, and EV-D68, a recent outbreak in United States in 2014^4^. EV71 causes cold-like symptoms, hand-foot-and-mouth disease (HFMD), encephalitis, acute flaccid paralysis, and death in children^1^,^5^,^6^. In addition, patients recovered from EV71 infection could have long-term neurological and cognition disorders^7^. EV71 has become a critical epidemic problem in recent years in Taiwan and Asia-Pacific area^8^,^9^. A user-friendly small animal model is an urgent need for EV71 research.

Previously, successful *in vivo* EV71 infection and pathogenesis can be achieved by intraperitoneal (i.p.) inoculation of adaptive viral strains into 1–3 day old ICR mice^10^,^11^. This model provided an *in vivo* platform for EV71 research. However, serially passaged adaptive strains of the RNA virus quasispecies are known to have undergone extensive variations and evolution, leading to diverse viral populations from the parental strain^10^,^12^. In addition, 1–3 day old newborns are very fragile to handle experimentally. Recently, many cellular receptors have been proposed for EV71 viral entry *in vitro*, including hSCARB2 (human Scavenger receptor class B), PSGL1 (P-selectin glycoprotein ligand 1), annexin II, and nucleolin^13^,^14^,^15^,^16^. It is important to demonstrate that these proposed receptors can indeed facilitate viral entry of EV71 *in vivo*. So far, human SCARB2 transgenic mouse is the only model shown to provide a functional role of hSCARB2 in the EV71 *in vivo* infection.

In one of the hSCARB2 transgenic mouse models, EV71 infection was successful at the age of 3-week-old, particularly when using a so-called Isehara strain of EV71 via an intracranial (i.c.) or intravenous (i.v.) route. Other viral strains were not as virulent and robust as the Isehara strain in this model. Because disease manifestations were assessed by combining ataxia, paralysis, and death into one single score^17^, it is difficult to compare the combined score to the conventional individual scores for paralysis and survival, without the inclusion of ataxia. In another hSCARB2 transgenic model^18^, no significant difference in the incidence of limb paralysis was observed between the hSCARB2 transgenic and non-transgenic control mice, when infected subcutaneously with EV71 genotypes B4 and C4. An approximately 2-fold difference in the paralysis rates was noted between transgenic and non-transgenic mice, when infected with genotypes C2 and B5. Another feature of this model is that only 1-day-old mice can be infected productively with EV71 genotype B. Newborns older than 1-day appeared to be rather resistant to EV71 infection and pathogenesis. Currently, there is no data available to side-by-side cross compare the phenotypes and susceptibility of various hSCARB2 Tg mouse models to EV71 infection. Therefore, careful choice of particular EV71 strains and particular mouse models could be critical for productive infection and pathogenesis.

Immunodeficient mouse models without bearing a hSCARB2 transgene, such as AG129, G129, NOD/SCID, and *stat-1* KO (*stat-1*^−/−^) mice, can also support efficient *in vivo* infection with EV71^19^,^20^,^21^. This result raises an issue whether hSCARB2 is the only receptor for EV71, and whether hSCARB2 plays a significant role in *in vivo* infection. The *stat-1* KO model is known to be deficient in interferon (IFN) signaling^22^. Similarly, G129 is deficient in IFN-gamma receptor, and AG129 is deficient in both IFN-alpha and IFN-gamma receptors^23^. The NOD/SCID model is deficient in both B and T cell maturation^24^. Overall, both adaptive and innate immunity are important against EV71 infection. Interestingly, different arms of the immune system predisposed the host infected with the same clinical isolate to different disease manifestations and tissue tropism^20^. Despite the fact that immune deficient mouse models can support *in vivo* infection with EV71, they generally require very high virus titer for efficient and productive infection (10^8^ pfu per mouse). In addition, newborn mice younger than 1-week after birth are required for susceptibility to experimental infection with clinical isolates of EV71. In contrast, the young age requirement for productive infection and pathogenesis was less stringent, or not observed, when mice were inoculated with mouse-adapted strains of EV71^10^,^21^.

In this study, we established an hSCARB2 transgenic mouse lineage and generated a hybrid mouse strain by crossbreeding the hSCARB2 transgenic mice with the *stat-1* KO mice (hSCARB2 Tg/*stat-1* KO). We found that this hybrid strain can be infected at 500-fold to 1000-fold lower titer of EV71 than the parental *stat-1* KO mice or the hSCARB2 transgenic mice. In addition, this hybrid strain can be infected in a robust manner at the age of 2-week old after birth. Infected hybrid mice exhibited streaking of nerve fibers positive for viral protein VP1 in both brain and spinal cord. Neuropathology and microglial amplification are evident by Nissl’s staining and immunohistochemistry (IHC) for IBA1, an inflammation marker of microglial cells. These hybrid mice also tend to develop an earlier onset of limb paralysis and death. Taken together, both hSCARB2 receptor and innate immunity are important determinants for EV71 infection and pathogenesis. A striking synergistic effect from hSCARB2 and the deficiency in innate immunity enables *in vivo* infection with different genotypes of EV71 at a 2-3 log lower dose of viral inoculation.

## Results

### Generation of a new animal model highly susceptible to EV71 infection

**Figure 1:**
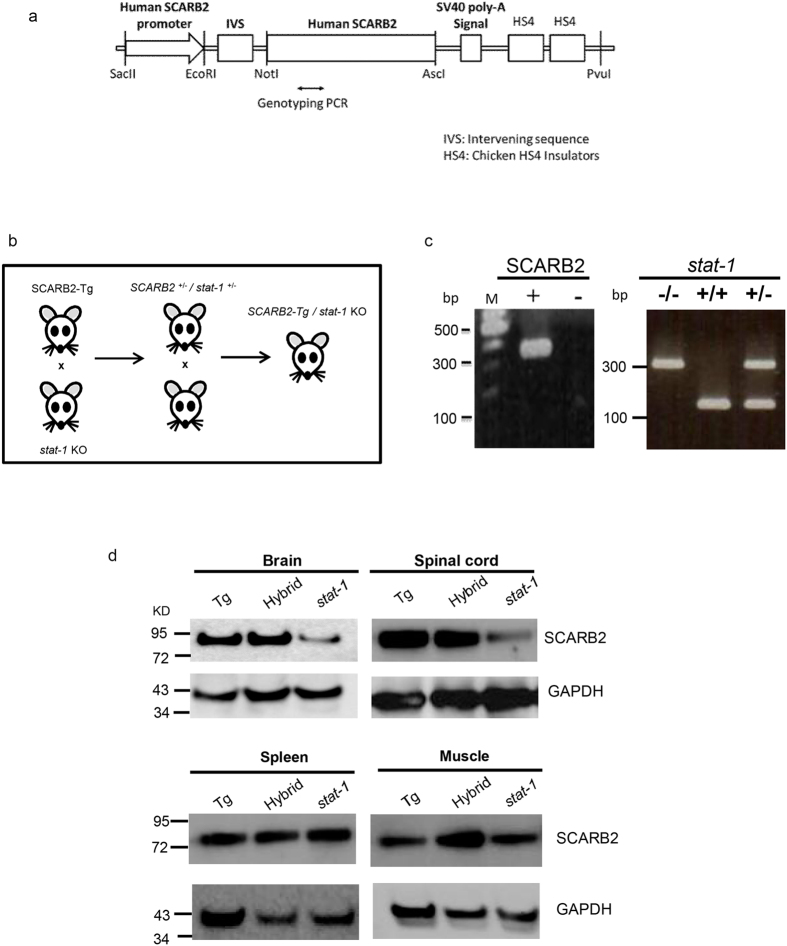
Generation of a hybrid mouse model SCARB2/*stat-1* KO by crossbreeding hSCARB2 transgenic mice and *stat-1* KO mice. (**a**) Human SCARB2 (hSCARB2) cDNA was cloned under its own native promoter in an SV40 expression vector. (**b**) The generation of homozygous hSCARB2^+/+^ transgenic mice was as detailed in M&M. The heterozygote strain of hSCARB2^+/−^/s*tat-1*^+/−^ mice were generated by crossing the *stat-1*^−/−^ and the hSCARB2^+/+^ parental mice. The hybrid strain hSCARB2^+/+^/*stat-1*^−/−^ was generated by crossing the heterozygote mice hSCARB2^+/−^/*stat-1*^+/−^ to each other. (**c**) Genotyping of parental and hybrid mouse strains was performed by PCR assay using genomic DNAs extracted from mouse tail. The transgene of hSCARB2 was screened by detection of a 369 bp PCR product using primers specific for hSCARB2. For the s*tat-1*^+/−^ heterozygote mice, 320 bp and 150 bp PCR products were used as markers for screening. The former indicates a mutant *stat-1* allele, while the latter indicates a wild type *stat-1* allele. (**d**) The expressions of hSCARB2 protein were compared among parental and hybrid mouse strains in brain, spinal cord, spleen and muscle by immunoblot via an anti-SCARB2 antibody. The weaker signals of the SCARB2 protein detected in *stat-1* KO mice reflect cross reactivity between human and mouse SCARB2 proteins to the anti-SCARB2 antibody.

We generated human SCARB2 transgenic mice using a hSCARB2 cDNA expression vector under the native promoter of hSCARB2 (Fig. 1a). The construction of this expression vector is as detailed in Materials and Methods. We screened the presence of the transgene hSCARB2 by using PCR primers specific for hSCARB2 (Fig. 1a). Generation of the homozygous hSCARB2 transgenic mouse is also detailed in Materials and Methods. We crossed the homozygous hSCARB2 transgenic mouse with *stat-1* KO mouse (*stat-1*^−/−^)^20^, and obtained heterozygote F1 with a genotype SCARB2^+/−^/*stat-1*^+/−^ (Fig. 1b). Self-crossing of this heterozygote hSCARB2^+/−^/*stat-1*^+/−^ mice generated the hybrid strain hSCARB2^+/+^/*stat-1*^−/−^. We first selected *stat-1*^−/−^ mice from the putative hybrid strains by using PCR primers specific for the mutant allele of *stat-1* (Fig. 1c). These identified *stat-1*^−/−^ mice were then mated with wild type C57BL/6. If all the progeny littermates were hSCARB2 positive, it would indicate that the hybrid mouse was the desired hybrid strain hSCARB2^+/+^/*stat-1*^−/−^. The expression of hSCARB2 protein can be detected in brain and spinal cord in homozygous hSCARB2^+/+^ mice and the hybrid strain hSCARB2^+/+^/*stat-1*^−/−^ by immunoblot analysis (upper panel, Fig. 1d). The weak signal in *stat-1*^−/−^ mice is from the endogenous mouse SCARB2 protein, which can cross-react with the anti-SCARB2 antibody.In contrast, we detected no apparent difference in SCARB2 protein expression in spleen and muscle (lower panel, Fig. 1d), which could be due to the low level expression of the exogenous hSCARB2 driven by the native promoter of hSCARB2 in spleen and muscle.

**Figure 2:**
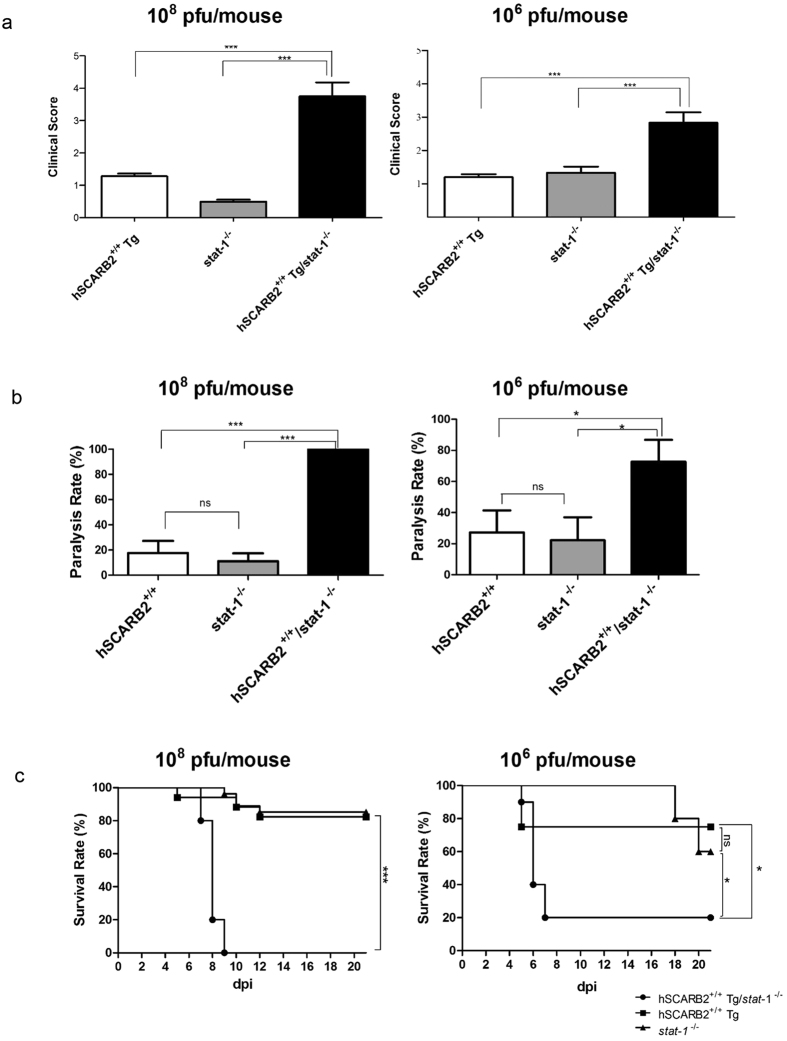
The hybrid mouse strain hSCARB2^+/+^/*stat-1*^−/−^ is most susceptible to infection and pathogenesis. One-week-old newborns of parental *stat-1*^−/−^, parental hSCARB2^+/+^ transgenic, and hybrid hSCARB2^+/+^/*stat*-1^−/−^ mice were i.p. infected with EV71 (genotype C2) at high titer (10^8^ pfu/mouse) and low titer (10^6^ pfu/mouse). Clinical scores, paralysis rates, and survival rates were compared among three different mouse strains. Disease manifestation and death were monitored daily post-injection. Clinical scores were defined as follows: 0, healthy; 1, hair loss, wasting, or ruffled hair; 2, limb weakness; 3, paralysis in only 1 limb; 4, paralysis in 2 to 4 limbs; 5, death. The hybrid strain exhibited the highest clinical score (**a**) paralysis rate (**b**) and the lowest survival rate (**c**). Numbers of infected mice were as summarized in Table 1.

To compare the susceptibilities among this hybrid strain and its parental strains, we inoculated one-week-old mice at high (10^8^ pfu/mouse) or low doses (10^6^ pfu/mouse) of EV71-(C2) (genotype C2) via ip route and monitored disease manifestation daily until 21 dpi. At both high and low doses of EV71, we observed significant differences in clinical scores (Fig. 2a) and paralysis rates (Fig. 2b), between the hybrid strain and its two parental strains. Similarly, significant difference in the survival rates was observed at either high or low doses of EV71 between the two parental and hybrid strains (Fig. 2c). The hybrid strain showed 100% death rate at 10^8^ pfu/mouse before 9 dpi, while the two parental strains showed less than 20% mortality on 21 dpi. At the lower dose of 10^6^ pfu/mouse, the difference in the death rate was apparent on 7 dpi (Table 1). At the lower dose of 10^5^ pfu/mouse, we detected in the hybrid strain approximately 57% of paralysis rate on 7 dpi, and all paralyzed mice died on 11 dpi. We detected no paralysis and death at 10^4^ pfu/mouse until 30 dpi. In addition to genotype C2, this hybrid strain can also be infected with genotype B5, albeit B5 required 10-fold higher inoculation titer than C2 for pathogenesis (Table 1). As summarized in Table 1, our results demonstrated that the hybrid strain hSCARB2^+/+^/*stat-1*^−/−^ is more susceptible to EV71 infection and pathogenesis than its two parental strains at either high or low doses of viral inoculation.

### Hybrid mice can be infected with clinical isolates of EV71 at an older age

**Figure 3:**
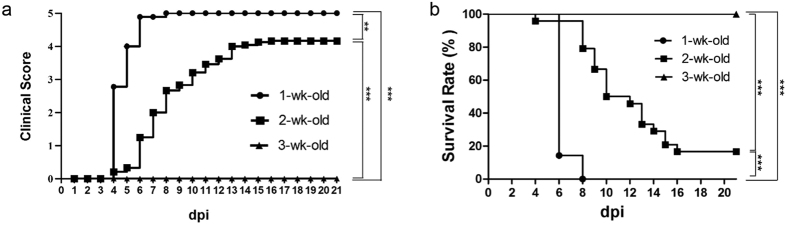
Young age is critical for the clinical score and survival rate in the hybrid mouse model infected with EV71. Clinical score (**a**) and survival rate (**b**) were compared in hybrid mice i.p. infected with EV71 (10^7^ pfu/mouse) at one-, two-, and three-week-old ages. Three-week-old hybrid mice showed no clinical symptoms and death. Numbers of infected mice were as summarized in Table 1.

Next, we compared 1-week, 2-week, and 3-week old mice of the hybrid strain for their pathogenesis upon EV71 infection at 10^7^ pfu/mouse. Disease manifestation was monitored daily up to 21 dpi. The result demonstrated that only 1- and 2-week old mice showed disease manifestation and death. The 3-week-old group displayed no detectable clinical score and exhibited 100% survival rate (Fig. 3a,b, Table 1). In the previous *stat-1*^−/−^ mouse model, only 1-week-old or younger neonates can be successfully infected with a clinical isolate of EV71 (genotype B5)^20^. Here, we demonstrated that 2-week-old hybrid mice can be infected with either genotype C2 or B5 in a highly robust manner.

### Streaking VP1 positive nerve fibers and inflammation in the CNS of infected hybrid mice

**Figure 4:**
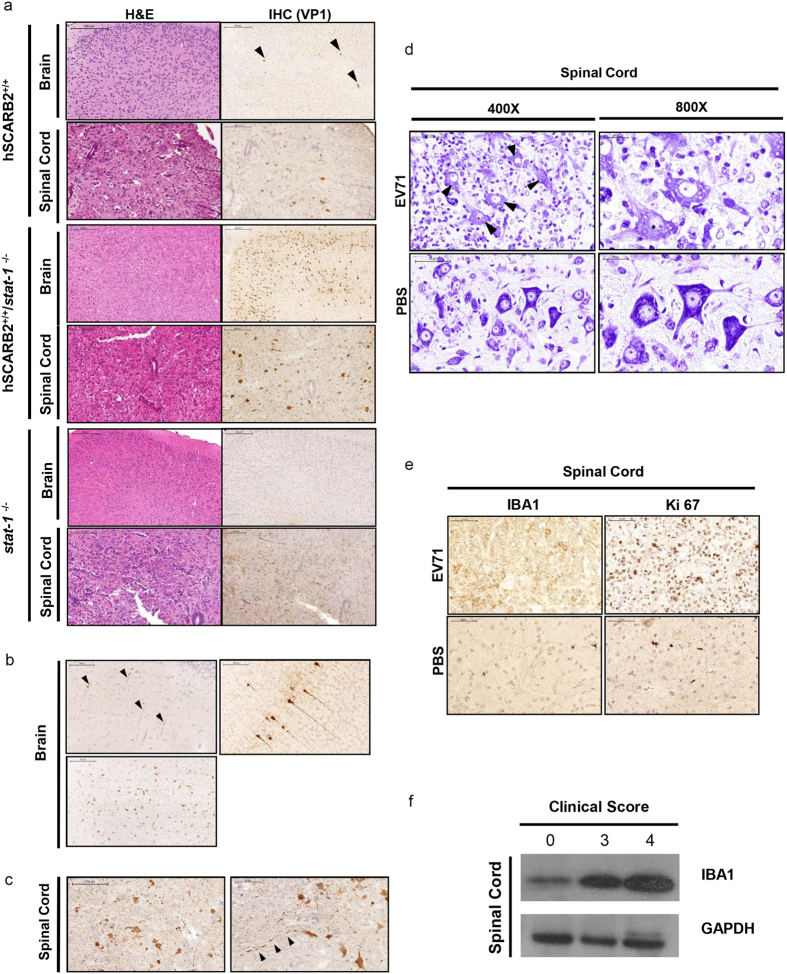
Histopathological examination of CNS sections in three different mouse models. (**a**) One-week-old of parental s*tat-1*^−/−^, parental hSCARB2^+/+^ transgenic, and hybrid hSCARB2^+/+^/*stat-1*^−/−^ mice were *in vivo* infected with 10^8^ pfu/mouse of EV71 (genotype C2) by i.p. route, respectively. Moribund mice were sacrificed. Paraffin-embedded sections of brain and spinal cord were examined with H&E stain at x100 and x200 magnifications, respectively (left panel). IHC staining detected the strongest expression of viral VP1 protein in brain and spinal cord in the hybrid mice (right panel). Black arrowheads in IHC of the transgenic hSCARB2^+/+^ mice indicate the VP1 positive signals. (**b**) Upper panel: Parallel streakings of VP1-positive nerve fibers and neuron cells in the mid-brain were detected by IHC staining in 2-week-old hybrid mice infected with EV71. Lower panel: A section from brain stem displayed VP1 positive signals. (**c**) Left panel: VP1 signal can be detected in lumbar section. Right panel: Streaking of nerve fibers was also detected in spinal cord sections (x400). Black arrowheads highlight the streaking nerve fibers. (**d**) Spinal cord sections were examined by Nissl Staining at 400x and 800x magnifications. Upper panel: EV71-infected hybrid strain mouse. Black arrowheads highlight abnormal neurons with extensive vaccuoles. Lower panel: PBS injected control mouse. (**e**) A microglial marker IBA1 was detected in spinal cord of infected hybrid strain mouse by immunohistochemistry (magnification at 400x in left panel). A cell proliferation marker Ki67 was strongly increased in infected spinal cord of hybrid strain mouse (magnification at 400x in right panel). (**f**) The expression of IBA1 protein in spinal cord appeared to correlate with the disease severity by immunoblot analysis. Each lane represents one spinal cord sample from each infected mouse with different clinical scores.

Previously, we observed virus-specific VP1 protein in brain and spinal cord of *stat-1*^−/−^ mice infected with EV71^20^. We compared here the histopathologies of brain and spinal cord among the parental and hybrid strains by H&E staining and immunohistochemistry (IHC) using anti-VP1 antibody. All strains of mice showed VP1 positive signals in brain and spinal cord (Fig. 4a), but not in muscle (Fig. S1). While there is no apparent difference in the H&E staining in sections of brain and spinal cord among these three different mouse strains, we detected higher density and stronger intensity of VP1 positive signals in neurons of the hybrid mice by IHC. These VP1 positive signals can be easily identified in retrosplenial granular cortex of the mid-brain in both hSCARB2^+/+^ and the hybrid hSCARB2^+/+^/*stat-1*^−/−^ mice. Interestingly, we observed an array of VP1-positive nerve fibers streaking in parallel to each other in mid-brain sections (upper panels, Fig. 4b). In addition, VP1-positive signals were detected in neuronal bodies from sections of brain stem (lower left panel, Fig. 4b). Similarly, VP1-positive neuronal bodies and streaking nerve fibers can be seen in sections of grey matter of the thoracic and lumbar vertebrate of spinal cord (arrowheads in Fig. 4c). Since lumbar spinal cord is known to be responsible for hindlimb movement, this result in Fig. 4c could explain the hindlimb paralysis observed in EV71 infected hybrid mice. By Nissl staining, we noted that neuronal cells in the spinal cord from EV71-infected hybrid mice lost the characteristic cytoplasmic blue dots (Nissl Bodies). In addition, these neuronal cells contained significant numbers of vacuoles as well as abundant numbers of small cells (Fig. 4d). Amplification of microglial cells is a known phenomenon of CNS inflammation and IBA1 is a known marker for microglial cells^25^. By immunohistochemistry (IHC) staining, IBA1 and Ki67 (a proliferation marker) proteins can be visualized in spinal cord sections from EV71 infected hybrid mice (Fig. 4e). We also detected increasing amount of IBA1 protein by Western blot analysis, when the clinical scores are increasing in EV71-infected hybrid mice (Fig. 4f). Taken together, our results suggest that both hSCARB2 receptor and *stat-1* mediated innate immunity are important for CNS infection and pathogenesis of EV71.

### Viral proteins and inflammatory cytokines were increased in CNS pathogenesis

**Figure 5:**
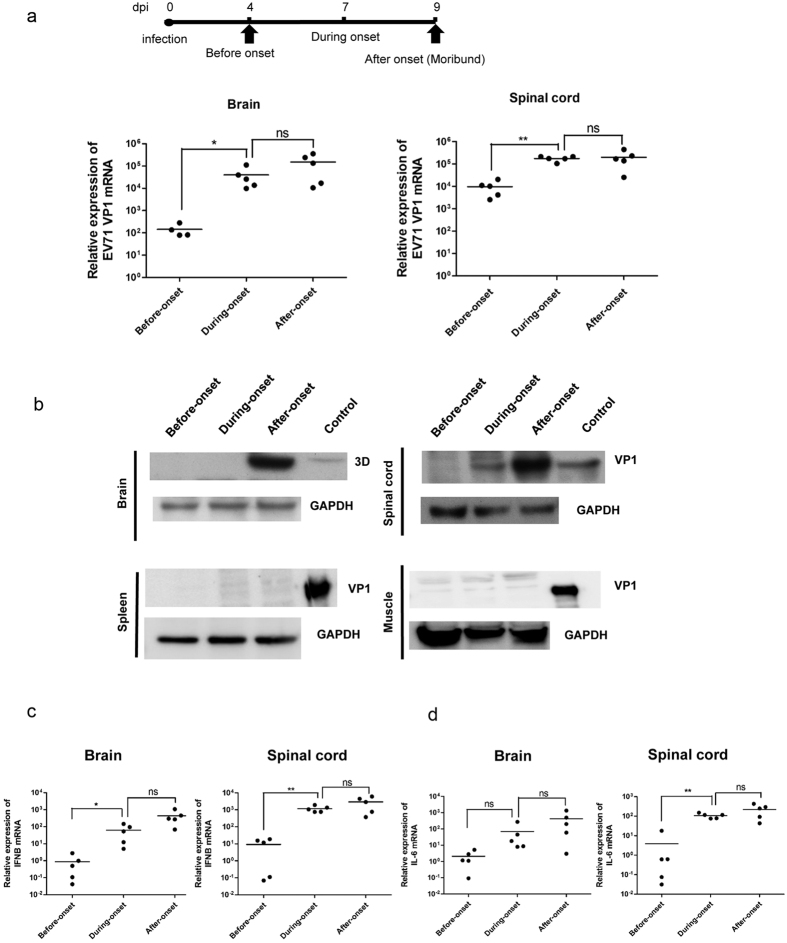
Expression profiles of EV71 specific RNA, protein and cellular cytokines in CNS of infected hybrid strain mice before, during, and after onset of limb paralysis. Two-week-old hybrid strain mice were infected with EV71 (genotype C2) at 10^7^ pfu/mouse by i.p. route. Brain, spinal cord, spleen and muscle were collected on dpi 4 (before-onset), dpi 7 (onset), and dpi 9 (after-onset). Extractions of viral RNA and protein were as described in Materials and Methods. (**a**) VP1 specific RNA in brain and spinal cord was both significantly increased at disease onset by RT-qPCR analysis. The results were normalized by GAPDH. (**b**) Viral proteins in brain (3D protein) and spinal cord (VP1 protein) peaked after disease-onset by immunoblot analysis. In contrast, no viral protein VP1 in spleen and muscle was ever detected throughout the entire time course. Lysates of EV71-infected RD cells were included as a positive control. (**c**) Expression of IFNB (IFN-beta) mRNA in brain and spinal cord was significantly increased at disease onset by RT-qPCR analysis. (**d**) Expression of IL-6 mRNA in spinal cord, but not in brain, was also increased at disease onset (Student’s *t* test). The results in (**c,d**) were normalized by GAPDH.

In our previous studies, we found elevations of a number of cytokines in EV71-infected mouse models^20^. Here, we focused on IL-6 and IFN-beta, since these two are important inflammatory cytokines expressed in EV71 infected patients^26^,^27^. To follow the time course of virus-host interactions post-inoculation, we compared the levels of EV71-specific RNA and protein, as well as IL-6 and IFN-beta before, during, and after disease onset (Fig. 5). VP1 specific RNA increased steadily from before-onset to after-onset in both brain and spinal cord by real-time RT-PCR analysis (Fig. 5a). VP1 protein followed a similar trend (Fig. 5b). We detected no EV71-specific RNA and protein (Fig. 5b) in spleen and muscle. We also monitored the profiles of inflammatory cytokines in brain and spinal cord. Unlike the expression patterns in spleen and muscle (Fig. S2), the patterns of IFN-beta (Fig. 5c) and IL-6 (Fig. 5d) in brain and spinal cord resembled those of VP1 RNA (Fig. 5a).

## Discussion

We generated here a novel mouse model containing both hSCARB2 transgene and *stat-1* KO. This new mouse model exhibited neuropathology in both brain and spinal cord, and thus recapitulated disease manifestations in EV71 natural infection in humans^8^. This model is more user-friendly than previous models in several ways, including the susceptibility to *in vivo* infection with EV71 of both genotypes C2 and B5 at a lower virus titer and an older age of mice.

In our hSCARB2 transgenic model and the *stat-1* KO model, mouse age younger than 1-week old is necessary for successful EV71 infection and pathogenesis. We experienced no successful infection and pathogenesis in 2-week old mice in either hSCARB2 transgenic model or the *stat-1* KO model. While we were able to infect the 2-week-old hybrid strain at 100% efficiency (10^7^pfu/mouse), we had no success in 20 inoculated hybrid mice at 3-week-old age (Table 1). We found no significant difference in hSCARB2 protein expression in brain and spinal cord by Western blot between 2-week-old and 3-week-old hybrid mice (Fig. S3). We also found no difference in the response of IFN-alpha, beta, and gamma in the brain and spinal cord between 2-week-old and 3-week-old hybrid mice inoculated with EV71 (Fig. S3). This age-dependent phenomenon of EV71 infection and pathogenesis is reminiscent of the age-dependent paralysis in a poliovirus receptor transgenic mouse model^28^,^29^. It remains to be investigated what kind of host factors could contribute to the age dependency, such as the neonatal development of the CNS or immune systems.

In the survival rate studies in Fig. 2c, the low dose inoculation (10^6^ pfu/mouse) of the two parental strains of mice appeared to have a higher tendency to develop death than the high dose (10^8^ pfu/mouse) group. In addition, both low dose and high dose inoculations developed onset of death at almost the same time in both hybrid mice and hSCARB2 transgenic mice. At present, we have no clear explanation for this phenomenon.

We observed no pathology in many tissues other than the CNS in the hybrid mice by H&E staining (Fig. S1). Nor could we detect any VP1 protein expression in muscle and spleen by IHC (Fig. S1). The lack of muscle tropism of EV71 in all three mouse strains could be related to the lower expression of hSCARB2 or the higher protective immunity in muscle. The most striking observation in the brain sections of EV71 infected hybrid mice is the streaking nerve fibers positive for the EV71 specific VP1 protein (Fig. 4). In addition, by H&E and Nissl stainings of spinal cord, we observed three major differences between the hybrid mice infected with EV71 and PBS. Upon infection with EV71, hybrid mice exhibited 1) extensive vacuole formation in neuronal cells; 2) microglial cell amplification; and 3) loss of Nissl bodies (rough ER) and reduced intensity in blue color (Fig. 4). Previous postmortem studies on fatal cases of EV71 infected children revealed severe involvement of brain stem and spinal cord, including inflammatory infiltrate of microglial cells^8^,^30^,^31^. Consistent with the neuropathology in brain and spinal cord, we detected significant increase in the mRNA levels of pro-inflammatory cytokine IL-6 and type 1 IFN at disease onset (Fig. 5c,d). In our previous studies, deficiency in IFN signaling in G129 mice and *stat-1* KO mice facilitated EV71 infection and pathogenesis^20^.

In summary, we established a new mouse model for EV71 *in vivo* infection and pathogenesis. This new model provides a user-friendly platform, which could facilitate the development of antiviral research in clinical medicine.

## Methods

### Ethics statement

All animal experiments were conducted under protocols approved by Academia Sinica Institutional Animal Care & Utilization Committee (ASIACUC Protocol number 13-12-622). Research was conducted in compliance with the principles stated in the Guide for the Care and Use of Laboratory Animals, National Research Council, 1996.

### Cell and virus preparation

Genotypes C2 (GenBank accession number: DQ060149) and B5 of EV71 were kindly provided by Dr. Mei-Shang Ho, IBMS, Academia Sinica, Taiwan^15^, and Changhua Christian Hospital, Changhua, Taiwan^20^, respectively. Both C2 and B5 of EV71 were clinical isolates from EV71 patients. Human rhabdomyosarcoma (RD) cells (ATCC CCL-136) were cultured in Dulbecco’s modified Eagle medium (DMEM; Gibco) with 10% fetal bovine serum (FBS; Hyclone) and 1% penicillin-streptomycin (Gibco) at 37 °C. For virus preparation, RD cells were cultured in T175 flask with 0.2% FBS and infected with EV71 at multiplicity of infection (MOI) of 0.01 at 37 °C for 24 hours. Virus was harvested by three cycles of freeze-and -thaw and centrifuged at 3000 × g at 4 °C for 30 minutes. Supernatant was concentrated by ultracentrifugation in Beckman SW28 rotors at 26000 rpm at 4 °C for 4–6 hours with 30% sucrose cushion and was resuspended in phosphate-buffered saline (PBS). EV71 titer was determined by plaque assy.

### Virus Titration

RD cell monolayer was cultured at a density around 5 × 10^5^ cells/well in 6-well plates (SPL life science). EV71 stock was 10-fold serially diluted with DMEM and RD cells were infected with virus at various dilutions. After 1 hour incubation, virus was removed from RD cells and covered with 4 mL DMEM containing 0.3% soft agar (Lonza) and 0.2% FBS at 37 °C for 72 hours. After 72 hours, RD cells were fixed by 3.7% formalin (Merck) for 1 hour at room temperature and the number of plaques was scored after crystal violet staining.

### Generation of hSCARB2 transgenic mouse

Human SCARB2 (hSCARB2) cDNA was amplified by PCR with primers NotI-SCARB2-F (GAGAGCGGCCGCCGGCCCGTGAGCGGCGCACA) and AscI-SCARB2-R (GAGAGGCGCGCCCCAAGCAAAGGCAATGTTTA) from a human SCARB2 cDNA clone (OriGene). The cDNA fragment was purified and cloned into a Pol’II-IVS expression vector (a kind gift from Dr. Ting-Fen Tsai, National Yang-Ming University, Taiwan) at NotI and AscI restriction sites. Human SCARB2 promoter (pSC2) sequences were amplified from the BAC clone RP-11-67-M24 (Invitrogen) by PCR with specific primers SacII-pSC2-F (GAGACCGCGGTTAGAGTCTGTGAAGAAAGCTCT) and EcoRI-pSC2-R (GAGAGAATTCCTCGCCGCAACCCCGCGAGG). The fragment of promoter sequences was purified and cloned into pol’II-IVS-hSCARB2 vector by SacII and EcoRI digestion. For the generation of transgenic mice, the pSC2-hSCARB2 expression vector was digested by SacII and PvuI, followed by purification and pronuclear injection in Transgenic Core Facility, Academia Sinica, Taiwan.

Homozygous hSCARB2 transgenic mice were established by first mating the F0 putative transgenic mice with wild type C57BL/6. The heterozygote F1 mice were crossed with each other to generate F2 mice. The F2 hSCARB2 transgenic mice were further crossed to wild type C57BL/6 mice to generate F3 mice. If F3 mice are 100% positive for the hSCARB2 transgene, then this F2 hSCARB2 transgenic line would be considered as a homozygous hSCARB2 transgenic lineage.

### Experimental infection

All mice were housed under specific-pathogen-free conditions in individual ventilated cages. One-to-three-week-old mice were infected with EV71 at the dose of 10^6^-10^8^ pfu/mouse via intraperitoneal route. Disease manifestations were monitored daily post-infection.

### Genotyping

Mouse DNA was extracted from mouse tail by using tissue DNA extraction kit (Thermo). Extracted DNA was used for PCR genotyping (My taq; Bioline). For SCARB2 Tg mice, specific PCR primers were: AGAGGGGAGACCCCTCGGGTG (forward) and AAAATA GGGAGAGATATCGGGCC (reverse). PCR product was 369 bp if the mouse carried human SCARB2 gene. For *stat-1*^−/−^ mice, three specific PCR primers were used. P1:GAGATAATTCACAAAATCAGAGAG, P2:CTGATCCAGGCAGGCGTTC, and P3:TAATGTTTCATAGTTGGATATCAT. For *stat-1*^−/−^ mice, PCR product should be 320 bp, while heterozygotes should be 320 and 150 bp.

### Histopathological and immunohistochemical (IHC) stain

The euthanized mice were perfused transcardially with PBS, followed by 10% neutral buffered formalin (CHIN I PAO CO., LTD, Taiwan). Tissue blocks were fixed in 10% neutral buffered formalin overnight. Fixed tissues were paraffin embedded, sliced, and stained with H&E by the Pathology Core Laboratory, IBMS, Academia Sinica. Xylene and ethanol were used for deparaffinization and rehydration. Retrieval buffer pH 6.0 (Dako) and endogenous enzyme blocker (Dako) were used for antigen retrieval and blocking steps. The slides were washed with PBS containing 0.1% Tween 20 (PBST) and incubated with rabbit anti-VP1 polyclonal antibody (PB7631, Abnova, Taiwan), rabbit anti-IBA1 polyclonal antibody (GTX 101495, Genetex, Taiwan), rabbit anti-Ki67 monoclonal antibody (GTX16667, Genetex, Taiwan), respectively. These slides were washed with PBST and incubated in secondary anti-rabbit antibody. DAB system (Dako) was used to visualize signals of antigens. Sections were counterstained with haematoxylin (J.T. Baker), and mounted with mounting reagent (MUTO Pure Chemicals). Images were scanned and presented via Pannoramic 250 FLASH (3DHISTECH Ltd).

### Real time reverse transcription–PCR

Infected mouse organs were harvested on Day 4 (before-onset), Day 6–7 (onset) and Day 8–10 (after-onset) post-infection. Organs were homogenized in PBS and aliquoted for RNA extraction. Tissue RNA extraction was conducted by following the WelPrep Tissue RNA Workbench protocol (Welgene). Complementary DNA was obtained by oligo dT-containing reverse transcriptase kit (Q-Amp MMuLV CDNA Synthesis Kit, Taiwan). Specific primers for VP1 were: forward: CTGGTAAAGGTCCAGCACTC, reverse: GGGAGGTCTATCTCTCCAAC; mouse GAPDH: forward: GTTCCTACCCCCAATGTG, reverse: CAACCTGGTCCTCAGTGTAG; IFNB: forward: GTACGTCTCCTGGATGAACTCC, reverse: CCTTTGCACCCTCCAGTAATAG; IL-6: forward: CCTCTCTGCAAGAGACTTCCAT, reverse: ACAGGTCTGTTGGGAGTGGTAT.

The results of VP1, IFNB and IL-6 expression were normalized by GAPDH, respectively.

### Immunoblotting

Radioimmunoprecipitation assay (RIPA) buffer was used to extract proteins from minced mouse tissue. Human SCARB2, VP1, EV71 3D protein, and GAPDH were detected by goat anti-SCARB2 (AF1966, R&D syst em) antibody, rabbit anti-VP1 polyclonal antibody (PB7631, Abnova, Taiwan), mouse anti-3D monoclonal antibody (GTX630193, Genetex, Taiwan) and rabbit anti-GAPDH polyclonal antibody (GTX100118, Genetex, Taiwan), respectively.

### Statistical analysis

Clinical scores of experimental mice were analyzed by one-way ANOVA. Survival rates of infected mice were analyzed with Log-rank test. Cytokine expression from 5 independent experiments was analyzed by Student’s *t* test. *P < 0.05; **P < 0.001; ***P < 0.0001.

## Additional Information

**How to cite this article**: Liou, A.-T. *et al*. A new animal model containing human SCARB2 and lacking *stat-1* is highly susceptible to EV71. *Sci. Rep*. **6**, 31151; doi: 10.1038/srep31151 (2016).

## Supplementary Material

Supplementary Information

## Figures and Tables

**Table 1:** Summary of disease manifestations in three different animal models.

Host Age	EV71 Genotype	Mouse Strains	Limb paralysis to death/total number of infected mice		
			10^8^ pfu/mouse	10^7^ pfu/mouse	^@^10^6^ pfu/mouse
1-wk	C2	SCARB2 Tg	3/17 (*11/17)	2/9	3/11
		Hybrid	5/5	9/9	8/11
		*stat-1* KO	3/27 (*7/27)	1/17	2/9
		wt C57BL/6	0/8	0/15	ND^#^
	B5	SCARB2 Tg	0/3	ND	ND
		Hybrid	10/12	2/6	ND
		*stat-1* KO	4/4	1/7	ND
2-wk	C2	SCARB2 Tg	ND	0/11	ND
		Hybrid	ND	20/24	12/15
		*stat-1* KO	ND	0/4	ND
	B5	SCARB2 Tg	1/4	ND	ND
		Hybrid	7/7	8/8	4/9
		*stat-1* KO	1/4	ND	ND
3-wk	C2	Hybrid	ND	0/20	ND

@Approximately 57% of hybrid mice can be infected with EV71-(C2) at 10^5^ pfu/mouse, but no mouse can be infected at 10^4^ pfu/mouse.

*Hindlimb weakness/total infected mice. Some mice with limb weakness did not die, and some recovered from paralysis.

^#^ND: not done.
