# Supplementary material for: A new animal model containing human SCARB2 and lacking stat-1 is highly susceptible to EV71
Source: Sci Rep. 2016 Aug 8;6:31151. doi: 10.1038/srep31151 (PMC4976353; doi:10.1038/srep31151)
Supplement: Supplementary Information [file srep31151-s1.pdf]

A new animal model containing human SCARB2 and lacking *stat-1* is highly susceptible to EV71

Authors: An-Ting Liou[1,2,3], Szu-Yao Wu[2,4], Chun-Che Liao[2], Ya-Shu Chang[2], Chih-Shin Chang[2,4], and Chiaho Shih[1, 2 *]

[1] Taiwan International Graduate Program (TIGP) in Molecular Medicine, National Yang-Ming University and Academia Sinica, Taipei, Taiwan

[2] Institute of Biomedical Sciences, Academia Sinica, Taipei, Taiwan

[3] Institute of Biochemistry and Molecular Biology, National Yang-Ming University, Taipei, Taiwan

[4]Institute of Microbiology and Immunology, National Yang-Ming University, Taipei, Taiwan

*To whom correspondence should be addressed

Mailing address: Institute of Biomedical Sciences, Academia Sinica, Taipei, Taiwan

Tel: 886-2-2652-3996; Fax: 8862-2652-3597; E-mail: cshih@ibms.sinica.edu.tw

Fig. S1

a

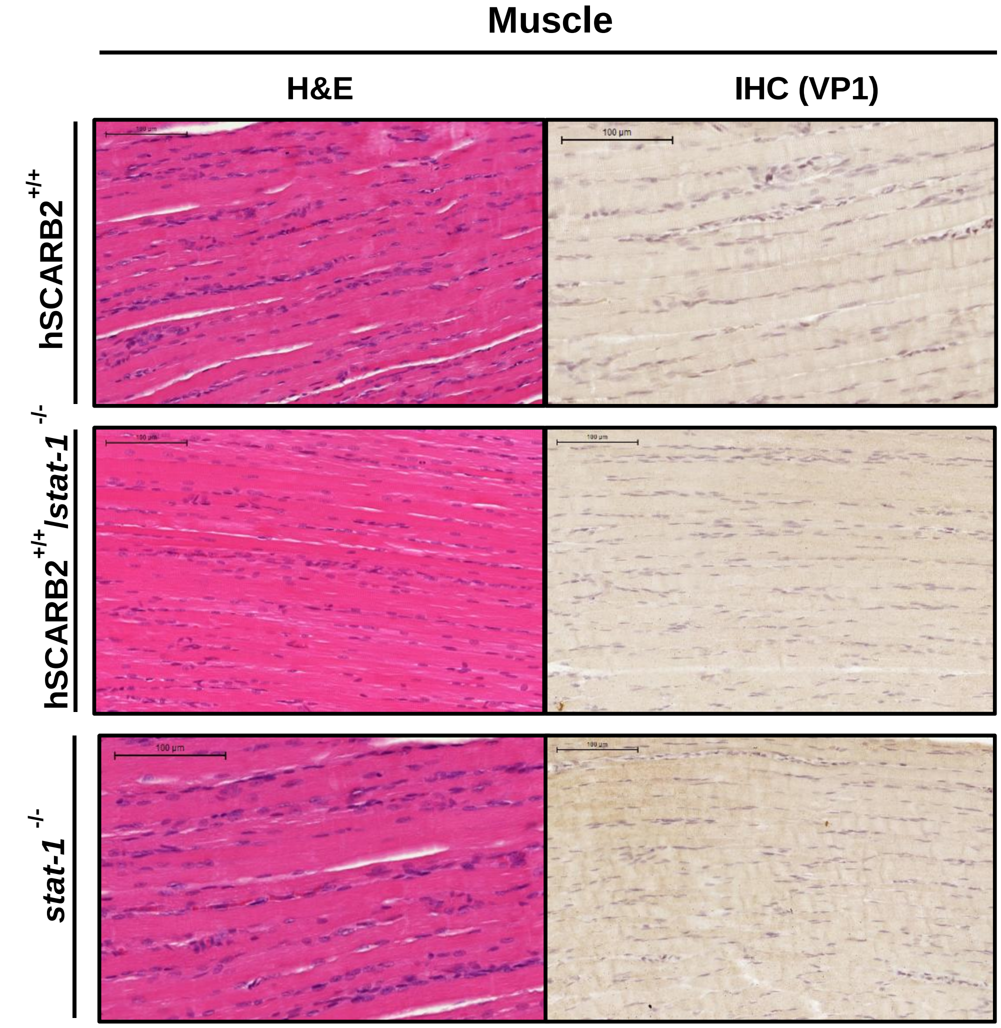
Muscle
H&E
IHC (VP1)
hSCARB2+/+
hSCARB2+/+/stat-1-/-
stat-1-/-
100 µm

b

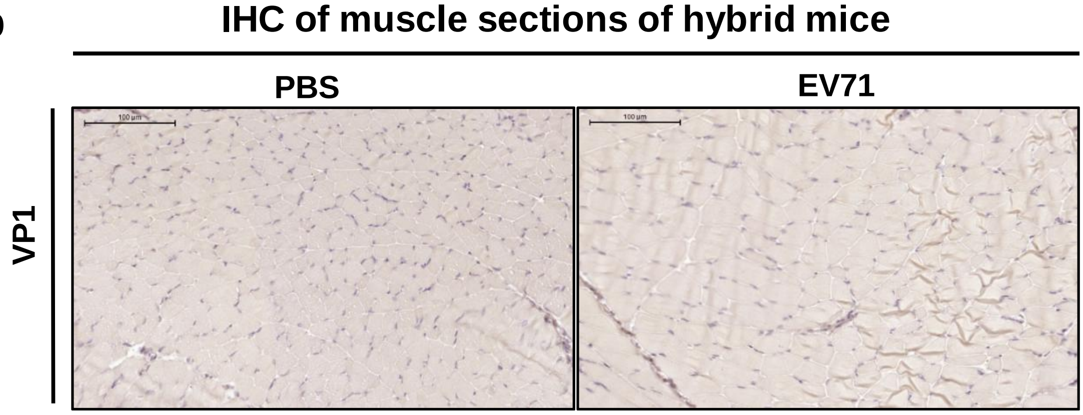
IHC of muscle sections of hybrid mice
PBS
EV71
VP1
100 µm

Fig. S1 Histopathological comparisons of sectioned muscle in parental and hybrid mice with or without EV71 infection.

(a) One-week-old of parental s*tat-1* $^{-/-}$, parental hSCARB2$^{+/+}$ transgenic, and hybrid hSCARB2$^{+/+}$/*stat-1* $^{-/-}$ mice were i.p. infected with 10^8 pfu/mouse of EV71 (genotype C2), respectively. Moribund mice were sacrificed. Paraffin-embedded sections of muscle were examined with H&E stain at x200 magnifications, respectively (left panel). IHC staining detected no viral VP1 protein expression in muscle in all strains of mice (right panel).

(b) One two-week-old hybrid hSCARB2$^{+/+}$/*stat-1* $^{-/-}$ mouse was i.p. infected with 10^7 pfu/mouse of EV71 (genotype C2). Moribund mice were sacrificed. IHC staining detected no expression of viral VP1 protein in hindlimb muscle in infected hybrid mice.

Fig. S2

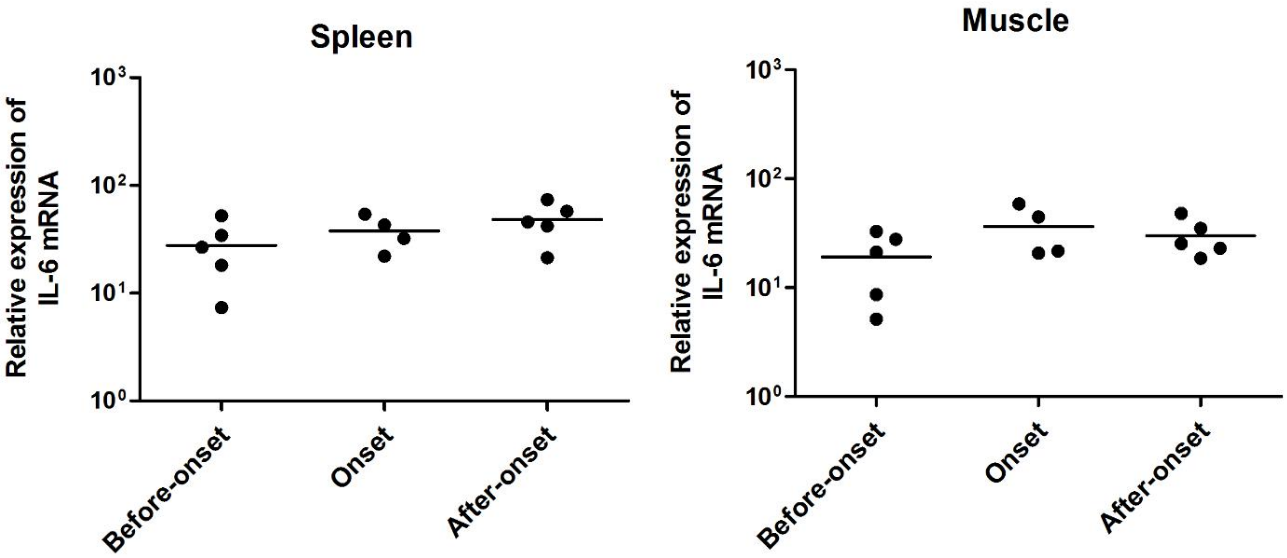

.

Fig. S2 IL-6 RNA expression in spleen and muscle in EV71 infected hybrid mice was measured by RT-qPCR..

No apparent difference in the expression of IL-6 mRNA was detected in spleen and muscle at different time points after EV71 inoculation.

Fig. S3

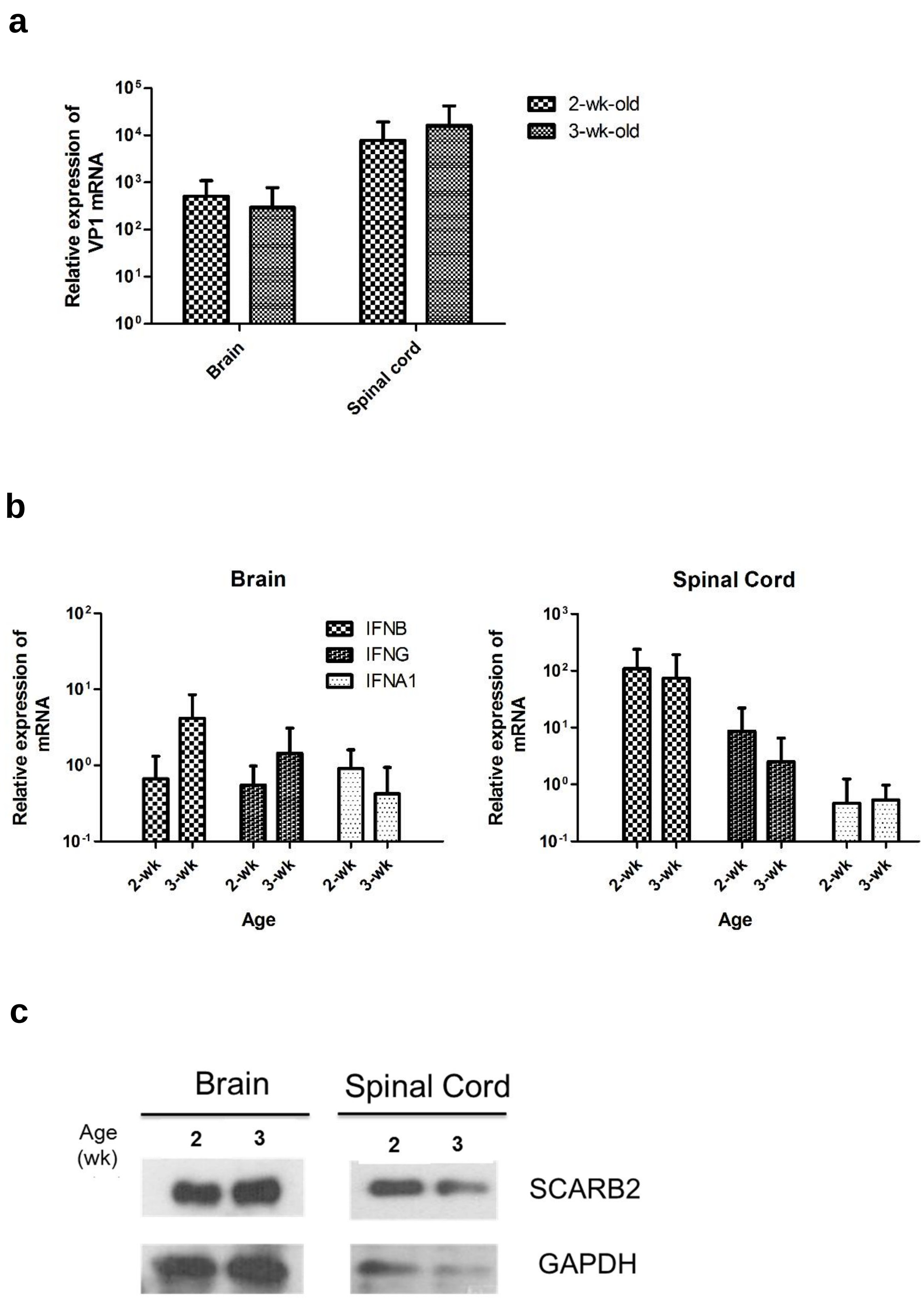
a
10⁵
10⁴
10³
10²
10¹
10⁰
Relative expression of VP1 mRNA
2-wk-old
3-wk-old
Brain
Spinal cord
b
Brain
Spinal Cord
Relative expression of mRNA
IFNB
IFNG
IFNA1
2-wk
3-wk
Age
c
Brain
Spinal Cord
Age (wk)
2
3
SCARB2
GAPDH

Fig. S3 Comparisons of VP1, hSCARB2 and interferon expressions between 2-week- and 3-week-old hybrid mice infected with EV71.

Brain and spinal cord RNAs were extracted from EV71 (C2)-infected 2-week-old mice (n=5) and 3-week-old mice (n=5) at 4 dpi (before disease onset). RT-qPCR was performed to compare RNA expression in brain and spinal cord between 2-week-old and 3-week-old groups. (a) Similar levels of VP1 specific RNA were detected. (b) Similar levels of IFNA1 (IFN-alpha), IFNB (IFN-beta), and IFNG (IFN-gamma) RNAs were detected. Student's *t* test was used to analyze the results and no significant difference between all comparisons. (c) No significant difference in hSCARB2 protein expression in brain and spinal cord was detected between 2-week- and 3-week-old mice by Western blot analysis. GAPDH was included as an internal control.
